# Unlike Physical Exercise, Modified Environment Increases the Lifespan of SOD1^G93A^ Mice However Both Conditions Induce Cellular Changes

**DOI:** 10.1371/journal.pone.0045503

**Published:** 2012-09-20

**Authors:** Yannick N. Gerber, Jean-Charles Sabourin, Jean-Philippe Hugnot, Florence E. Perrin

**Affiliations:** 1 INSERM U1051, Institute for Neurosciences of Montpellier, Pathologies Sensorielles, Neuroplasticité et Thérapies, Saint-Eloi Hospital, Montpellier, France; 2 IKERBASQUE Basque Foundation for Science, Bilbao, Spain; 3 Integrative Biology of Neurodegeneration, Neuroscience Department, University of the Basque Country UPV/EHU, Bilbao, Spain; Baylor College of Medicine, Jiao Tong University School of Medicine, United States of America

## Abstract

**Background:**

Amyotrophic lateral sclerosis (ALS) is characterized by a gradual muscular paralysis resulting from progressive motoneurons death. ALS etiology remains unknown although it has been demonstrated to be a multifactorial disease involving several cellular partners. There is currently no effective treatment. Even if the effect of exercise is under investigation for many years, whether physical exercise is beneficial or harmful is still under debate.

**Methods and Findings:**

We investigated the effect of three different intensities of running exercises on the survival of SOD1^G93A^ mice. At the early-symptomatic stage (P60), males were isolated and randomly assigned to 5 conditions: 2 sedentary groups (“sedentary” and “sedentary treadmill” placed on the inert treadmill), and 3 different training intensity groups (5 cm/s, 10 cm/s and 21 cm/s; 15 min/day, 5days/week). We first demonstrated that an appropriate “control” of the environment is of the utmost importance since comparison of the two sedentary groups evidenced an 11.6% increase in survival in the “sedentary treadmill” group. Moreover, we showed by immunohistochemistry that this increased lifespan is accompanied with motoneurons survival and increased glial reactivity in the spinal cord. In a second step, we showed that when compared with the proper control, all three running-based training did not modify lifespan of the animals, but result in motoneurons preservation and changes in glial cells activation.

**Conclusions/Significance:**

We demonstrate that increase in survival induced by a slight daily modification of the environment is associated with motoneurons preservation and strong glial modifications in the lumbar spinal cord of SOD1^G93A^. Using the appropriate control, we then demonstrate that all running intensities have no effect on the survival of ALS mice but induce cellular modifications. Our results highlight the critical importance of the control of the environment in ALS studies and may explain discrepancy in the literature regarding the effect of exercise in ALS.

## Introduction

Amyotrophic lateral sclerosis (ALS) is a chronic neurodegenerative disease characterized by a selective motoneurons death in the motor cortex, brainstem and spinal cord that leads to progressive muscular paralysis. Pathogenesis of motoneuron degeneration in ALS and mechanisms of selective vulnerability are still largely unknown although it has been demonstrated that ALS is a complex multifactorial disease (protein misfolding, glutamate-mediated excitotoxicity, oxidative stress, impaired axonal transport…) that involves, besides neurons, several cellular partners such as glial and muscle cells (for reviews see [1], [2]). Thus, there is growing interest about whether the death of motoneurons is cell autonomous or mediated by non-neuronal cells [3], [4]. A better understanding of the role of these partner cells has resulted in the recognition of the important role played by astrocytes and microglia in ALS (for review see [5]). Indeed, the presence of reactive astrocytes and microglia is a hallmark of ALS [6], [7].

With the aim of translation to the clinic, and since epidemiological reports suggested that an active lifestyle may be associated with an increased incidence of ALS [8], several studies investigated the effect of physical exercise on ALS patients and on animal models of ALS (for review see [9], [10]. Whether physical exercise is beneficial or detrimental is still a matter of debate. On the one hand, moderate exercise is reported to increase the life span of several mouse models of motoneuron disease such as SOD1^G93A^ mice [11], [12], of spinal muscular atrophy such as survival motor neuron2/SMN2 mice [13] and progressive motor neuronopathy/pmn mice [14]. On the other hand, high intensity exercise was reported to have no effect [15] or to be detrimental [16] to the survival of SOD1^G93A^ mice.

In order to reconcile these disparate results, we investigated the effect of three different intensities of running-based exercise on the survival of SOD1^G93A^ mice. As a pre-requisite we evaluated two different sedentary groups. Surprisingly, we demonstrate that a slight daily modification of the environment extend the lifespan of SOD1^G93A^ mice by 11.6%. Moreover, increased lifespan is accompanied with motoneuron survival and with an increase in glial cells reactivity. When compared with the appropriate control group, we demonstrate that running exercise also induces cellular modifications but, whatever its intensity, is not associated with a variation in lifespan.

These results not only highlight the importance of the environment in ALS but may also explain the discrepancy in the literature about running-based exercise, due to an inappropriate choice of control groups.

## Results

### Unlike Exercise, Modified Environment Increases the Lifespan of SOD1^G93A^ Mice

**Figure 1:**
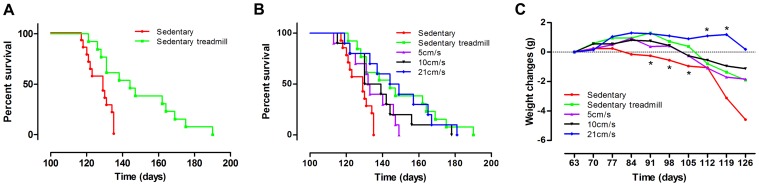
Effects of modified environment and running-based training intensities on SOD1^G93A^ mice survival. (**A**) – Kaplan-Meier curves of “sedentary” (n = 14) and “sedentary treadmill” (n = 13) SOD1^G93A^ mice (log rank test, **p = 0.0015). (**B**) – Kaplan-Meier curves of “sedentary” (n = 14), “sedentary treadmill” (n = 13), 5 cm/s (n = 10), 10 cm/s (n = 10) and 21 cm/s (n = 10) groups. (**C**) – Body weight changes in the “sedentary”, “sedentary treadmill” and 3 exercised groups from P60 to end of life. Statistics: t-test; *p<0.05. All statistics were done using the sedentary treadmill group as reference.

In a first step, to minimize experimental bias, we controlled animal handling parameters by a survival analysis of two sedentary groups of SOD1^G93A^ male mice. The two groups differed by only one parameter: mice from the “sedentary” group constantly remained alone in their cage whereas “sedentary treadmill” mice were placed on the inert treadmill for 15 minutes a day and 5 days a week ***(Figures S1 and S2)***. Surprisingly, the median survival of “sedentary treadmill” mice (144 days) exceeded that of the sedentary group (129 days) by 15 days (corresponding to an 11.6% increase), and Kaplan–Meier survival statistics revealed a significant group difference in survival time (p = 0.0015) ***(*** ***Figure 1A*** ***, Figure S3A)***.

In a second step, to assess the effect of running-based exercise on the survival of SOD1^G93A^ mice, we subjected different animal groups to three regimens of regular exercise (5, 10 and 21 cm/s; 15 minutes a day, 5 days a week). The exercise protocol started at the early-symptomatic stage (60 days of age) and ended when mice were unable to maintain the exercise regimens. Median survivals were of 132.5, 134.5 and 146.5 days for the 5, 10 and 21 cm/s respectively. When compared with the appropriate control, i.e. “sedentary treadmill” group, Kaplan-Meier survival statistics evidenced no significant difference for any of the trained groups ***(*** ***Figure 1B*** **,** ***Figure S3A)***. However comparisons amongst exercised mice revealed that the highest intensity of exercise group (21 cm/s) had an increased median survival than the lowest intensity group (5 cm/s) (146.5 vs 132.5 p = 0.0439).

Over the course of the disease, all ALS mice lost weight as a result of muscular atrophy ***(*** ***Figure 1C*** ***)*** *.* We thus monitored body weight changes in all groups (sedentary and exercise) from the beginning of the training protocol to the death of the mice. Animals subjected to the “sedentary treadmill” protocol maintained their body weight longer than “sedentary” mice (respectively until day 105 (p = 0.017) vs day 91 (p = 0.0102)). Mice subjected to high intensity exercise (21 cm/s) conserved their body weight longer than the “sedentary treadmill” group (respectively until day 119 (p = 0.026) vs day 112 (p = 0.0359)) ***(*** ***Figure 1C*** ***)***.

### Motoneurons are Preserved in “Sedentary Treadmill” and Exercised Animals

Differences in lifespan between the two sedentary groups suggest possible cellular modifications resulting from the “sedentary treadmill” protocol. Considering that body mass differences between “sedentary” and “sedentary treadmill” ALS mice started at 90 days of age, we characterized cellular elements of the lumbar spinal cord and the *gastrocnemius-soleus-plantaris* complex in the two groups at P90. Similar analyses were then done for all trained groups.

**Figure 2:**
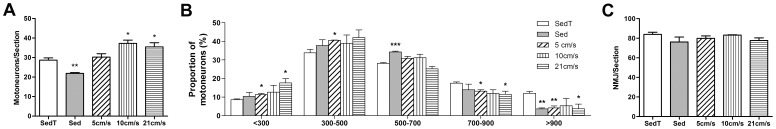
Motoneurons and neuromuscular junctions in “sedentary” and “sedentary treadmill” mice at P90. (**A**) – Quantification of motoneurons in the lumbar spinal cord of “sedentary”, “sedentary treadmill” and exercised animals at P90 (hematoxylin coloration). (**B**) – Measurement of motoneuron cell body area in all groups at P90 (in µm^2^). (**C**) – Quantification of the neuromuscular junctions in the *gastrocnemius-soleus-plantaris* complex using the Karnovsky and Roots enzymatic method in sedentary and exercised groups at P90 (Statistics: t-test; *p<0.05, **p<0.01, ***p<0.001; n = 3 in each group) Sed = sedentary, SedT = sedentary treadmill. All statistics were done using the sedentary treadmill group as reference.

We first quantified the number of lumbar motoneurons. We showed that “sedentary treadmill” mice exhibit a significant increase in motoneuron survival as compared to the “sedentary” animals (28.69±1.164 vs. 21.90±0.4089) ***(*** ***Figure 2A*** **,** ***Figure S3B)***. Moreover, this neuroprotection is correlated to a significant preservation of the mean cell body area in the “sedentary treadmill” mice as compared to the “sedentary” group ***(*** ***Figure 2B*** **,** ***Figure S3B)***. Indeed, analysis of the mean motoneuron area, demonstrate a strong preservation of large motoneurons in the “sedentary treadmill” animals (***Figure 2B***). Motoneurons quantification in the trained groups highlighted a better survival in the 10 and 21 cm/s (37.28±1.655 and 35.47±2.119) groups as compared to “sedentary treadmill” mice (28.69±1.164) ***(*** ***Figure 2A*** **,** ***Figure S3B)***. However, motoneurons mean cell body area was decreased in the 5 and 21 cm/s groups as compared to “sedentary treadmill” animals ***(*** ***Figure 2B*** **,** ***Figure S3B)***. The cell body area may indeed be considered as a marker of the physiological stage of motoneurons, indeed, before death motoneurons shrink. At end stage no significant difference was observed in the number of motoneurons *(data not shown)*.

Modifications of motoneuron survival in the lumbar spinal cord prompted the examination of neuromuscular junctions (NMJ) in the *gastrocnemius-soleus-plantaris* complex at 90 days of age. We did not notice any statistically significant differences in NMJ’s number between all groups (Sed: 76.15±5.032; SedT: 83.99±2.172; 5 cm/s: 79.9±2.5; 10 cm/s: 83.21±0.3685; 21 cm/s: 77.61±2.778).

### “Sedentary treadmill” Animals Harbor a Strong Enhanced Glial Reactivity

**Figure 3:**
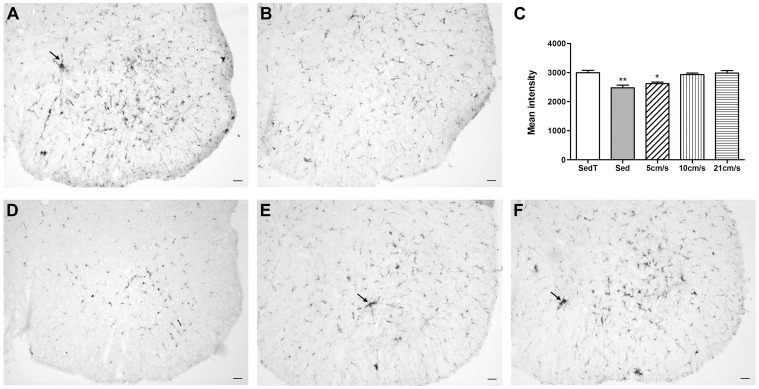
Microglial activation in sedentary and exercised animals. (**A, B, D, E & F**) – Photographs of Iba1 immunohistochemical staining of P90 lumbar spinal cord sections from “sedentary treadmill” (**A**), “sedentary” (**B**), 5 cm/s (**D**), 10 cm/s (**E**) and 21 cm/s (**F**) animals respectively. (**C**) – Quantification of the mean intensity of labeling in the ventral gray matter. Statistics: t-test; *p<0.05, **p<0.01. Scale bars**:** 50 µm. Sed = sedentary, SedT = sedentary treadmill. In panels A, E and F arrow point to of high density “packs” of microglia. All statistics were done using the sedentary treadmill group as reference.

**Figure 4:**
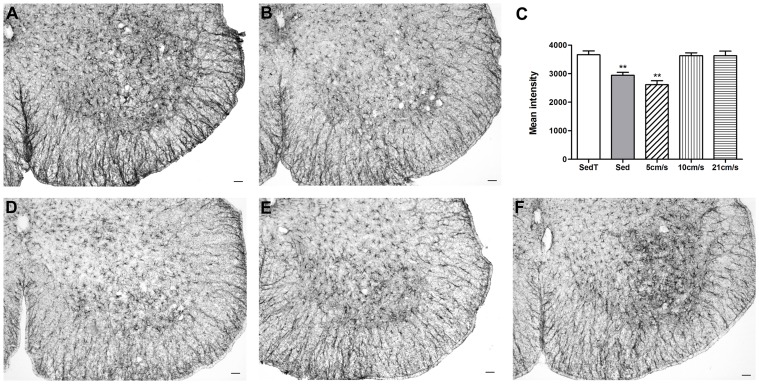
Astrocytes reactivity in sedentary and exercised animals. (**A, B, D, E & F**) – Photographs of GFAP immunohistochemical staining of P90 lumbar spinal cord sections from “sedentary treadmill” (**A**), “sedentary” (**B**), 5 cm/s (**D**), 10 cm/s (**E**) and 21 cm/s (**F**) animals respectively. (**C**) – Quantification of the mean intensity of labeling in the ventral gray matter. Statistics: t-test; **p<0.01. Scale bars**:** 50 µm. Sed = sedentary, SedT = sedentary treadmill. All statistics were done using the sedentary treadmill group as reference.

**Figure 5:**
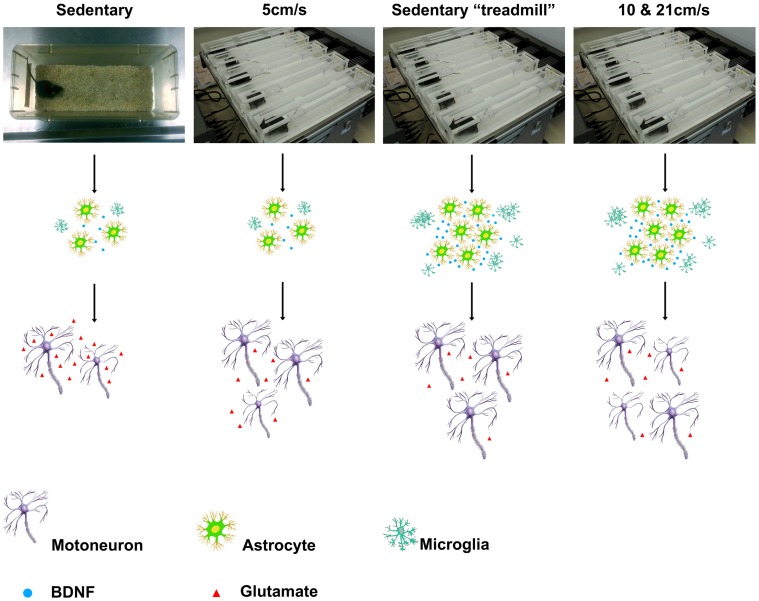
Schematic representation of the effect of modified environment and exercise on glial reactivity and motoneurons preservation in the SOD1^G93A^ mice. At 90 days of age, sedentary treadmill mice and mice submitted to moderate (10 cm/s) or high exercise (21 cm/s) regimens harbor a marked increase in astrogliosis and a strong microglial reactivity as shown by the presence of high density “packs” of microglia. Conversely, sedentary and 5 cm/s animals show limited astrogliosis and no microglia “packs”. Moreover, modifications observed in the glial compartment correlate with changes in motoneurons survival and morphology. Whereas a dramatic decrease in motoneuron number and size is observed in sedentary mice, exposure to exercise or modified environment induces motoneuron preservation. However, effect on motoneurons preservation depends on the exercise regimen. Indeed, when compared to sedentary treadmill animals, 5 cm/s mice show a preserved number of motoneurons but an altered phenotype and a reduced soma size. On the other hand, 10 and 21 cm/s trained animals present a better preservation of motoneurons number but show an altered phenotype as compared to the sedentary treadmill group.

There are growing evidences of the involvement of glial cells in ALS. To detect glial correlates of motoneuron survival in the “sedentary treadmill” group we investigated possible modifications of microglia and astrocytes at 90 days of age and at the end stage. To evaluate gliosis, we used an immunohistochemical approach to detect the microglial marker ionized calcium binding adaptor molecule 1 (Iba1) and the astrocytic marker glial fibrillary acidic protein (GFAP). At 90 days of age, we observed a marked increase in Iba1 in the ventral grey matter of “sedentary treadmill” animals as compared to the “sedentary” group. The mean intensity of labeling was indeed about 20% higher (2478±88.6 in the “sedentary” vs. 2999±82.32 in the “sedentary treadmill”) ***(*** ***Figures 3*** ***A–C, Figure S3C)***. Moreover, we observed clear morphological evidence of microglial activation in the ventral spinal horns of “sedentary treadmill” animals as compared to “sedentary”. Indeed, microglia from “sedentary” animals displayed a large soma associated with thick ramified processes that are characteristics of early microglial activation whereas “sedentary treadmill” mice harbored microglia with large soma and reduced processes complexity. In addition, the ventral lumbar spinal horns of “sedentary treadmill” animals presented local increased microglia densities ***(*** ***Figure 3A*** ***, arrow)***, characteristics of an inflammatory state. In parallel, GFAP staining was increased by ≈1.25 fold in the ventral grey matter of “sedentary treadmill” group as compared to the “sedentary” (2944±104.2 in the “sedentary” vs. 3666±136.2 in the “sedentary treadmill”) ***(*** ***Figures 4*** ***A–C,*** ***Figure 5*** ***, Figure S3D)***. Conversely, at end stage, we did not observe any difference in GFAP or Iba1 staining between the two sedentary groups *(Data not shown)*.

### Effects of Exercise Regimens on Glial Reactivity

As for the sedentary groups we analyzed glial components at 90 days of age. The mean intensity of Iba1 microglia labeling was decreased in the 5 cm/s group as compared to “sedentary treadmill” mice (2621±54.49 vs 2999±82.32) (***Figure 3A, C–D*** **,** ***Figure S3C***). Moreover, microglia morphology of the 5 cm/s animals was similar to the one of the sedentary group (***Figure 3B&D***). For both, intensity of labeling and morphology, microglia were similar in the “sedentary treadmill”, the 10 and 21 cm/s groups (2999±82.32, 2934±52.28 and 2985±89.08 respectively) (***Figure 3A, C, E&F*** **,** ***Figure S3C***).

Astrocytes behaved similarly, indeed, the mean intensity of GFAP astrocyte labeling was decreased in the 5 cm/s group as compared to “sedentary treadmill” mice (2615±140.7 vs 3666±136.2) (***Figure 4A, C–D*** **,** ***Figure S3D***). The labeling intensity of GFAP was similar in the “sedentary treadmill”, the 10 and 21 cm/s groups (3666±136.2, 3631±102.6 and 3631±161.1 respectively) (***Figure 4A, C, E&F*** **,** ***Figure 5*** **,** ***Figure S3D***).

All together, these results demonstrate that placing the SOD1^G93A^ mice on an inert treadmill for 15 minutes, 5 days a week leads to an increase in the lifespan of the animals, which is correlated with motoneuron preservation and a markedly enhanced glial reactivity. When compared to the proper control i.e. “sedentary treadmill” animals, none of the 3 tested exercise intensities induced a modification in lifespan. However, we observed a preservation of motoneurons in the 10 and 21 cm/s exercised mice and a decrease in microglia and astrocytes for the 5 cm/s trained mice (***Figure 5***).

## Discussion

The initial aim of this study was to carefully evaluate the impact of different running intensities on the survival of male SOD1^G93A^ mice. For that purpose, we chose three different running regimens i.e. 5, 10 and 21 cm/s. At these speeds, mice reach 65, 70 and 80% of their maximal oxygen consumption respectively (for review see [17]). To evaluate the effect of exercise *per se,* we first compared two control sedentary groups and showed that placing the mice on an inert treadmill is sufficient to induce a mean survival increase of 11.6%. This result can be compared with previous studies on central nervous system pathologies demonstrating the effect of the environment on the progression of the disease [18]. We showed that increased survival of “sedentary treadmill” animals is associated with a longer preservation of the body weight. This may reflect a better muscle mass preservation and is consistent with the results of Stam et al. that demonstrated that an enriched environment can delay the loss of motor function in SOD1^G93A^ mice [19]. It is of interest to note that the 21 cm/s trained animals also maintain their body weight longer than other groups but without increased lifespan. This is most likely due to a gain in muscular mass induced by the exercise. This major difference in survival between control groups may explain previous outcome discrepancies on the effect of exercise. Indeed, when compared with the appropriate control group i.e. “sedentary treadmill” mice, we did not observe any modification in the lifespan of SOD1^G93A^ that underwent three intensities of running-based training. Our results thus confirm that high intensity exercise (21 cm/s) has no effect on the survival of SOD1^G93A^ mice as reported in a recent study that also used proper control mice [20]. Moreover, our study extends this result to lower training intensities (5 and 10 cm/s). However, amongst the trained groups, we observed a significant increase in survival between mice subjected to the highest intensity of exercise regimen (21 cm/s) and those subjected to the lowest one (5 cm/s). This may reflect a slight intensity-dependent effect of exercise that is not sufficient enough to induce a lifespan modification when compared to the accurate control (i.e. the “sedentary treadmill” group). To avoid an additional bias due to uncontrolled physical activity on the exercise level, we did not check motor performance over the course of the experiments. We have indeed previously shown that motor tests (rotarod, grip strength and grasping onto a grid) constituted by themselves a low intensity exercise in a mouse model of motoneuron disease [14].

To tentatively correlate differences in survival induced by a slight modification of the environment with cellular alterations and to further analyze possible effects of exercise at the cellular level, we carried out a histological characterization of the lumbar spinal cord and the *gastrocnemius-soleus-plantaris* complex.

Analysis of the spinal cellular partners at 90 days of age demonstrated higher motoneuron number preservation in the “sedentary treadmill” group when compared to sedentary animals. Mice subjected to moderate (10 cm/s) or high (21 cm/s) exercise intensity presented a better motoneuron survival than animals subjected to 5 cm/s-exercise or the “sedentary treadmill” groups ***(*** ***Figure 5*** ***)***. Moreover, “sedentary treadmill” mice had a largest motoneuron soma (motoneuron enrichment with a soma>700 µm^2^, ***Figure 2B*** **,** ***Figure S3B***) than exercised animals which may reflect a more advanced “dying-state” of motoneurons in trained groups. Thus, even motoneuron number was higher in trained animals, it most likely reflect only a slight delay in degeneration. This observation suggests that even if the 3 intensities of exercise fail to significantly modify SOD1^G93A^ survival, moderate and high exercise intensities may delay motoneurons death.

Conversely, we did not find any difference in NMJ numbers between all groups but we cannot exclude that NMJ of “sedentary treadmill” and/or trained mice present a better functionality. These results are thus in favour of a central (spinal cord) initiation of neuroprotective mechanisms rather than the result of a centripetal muscular influence.

At 90 days of age, analysis of the glial compartment allowed us to distinguish two groups according to their glial response. “Sedentary” and 5 cm/s animals showed weak microglia and astrocytes reactivity whereas “sedentary treadmill”, 10 and 21 cm/s mice harbor strong astrogliosis and a marked increase of microglial activation ***(*** ***Figure 5*** ***)***.

In particular, we evidenced an increase in GFAP staining in the lumbar ventral spinal grey matter of the “sedentary treadmill” group as compared to the “sedentary” one. This result is consistent with the finding that an enriched environment (EE) increases astrocytes number and GFAP expression in healthy and pathological conditions [21], [22], [23]. Moreover, in a model of Parkinson’s disease, EE-induced astrogliosis is accompanied by a decrease in neuronal loss [21]. Moreover, Li et al. have demonstrated in rats that running exercise increases astrocytes number in cortex and striatum, which is consistent with the strong astrogliosis observed in the 10 and 21 cm/s groups [24].One can hypothesize that the potential neuroprotective effect of astrocytes results from complex mechanisms.

EE and exercise stimulate the production of several growth factors. Amongst those, brain-derived neurotrophic factor (BDNF) level is increased in the dentate gyrus of animals housed in enriched conditions [25]. Even if “sedentary treadmill” conditions cannot be strictly considered as an enriched environment we can conjecture that the slight modification of “sedentary treadmill” mice daily environment is sufficient to induce similar effects. Moreover, Neeper et al. have shown that BDNF is also induced after running exercise in the hippocampus, the cortex and the cerebellum [26].In addition, BDNF is known for its beneficial effects, in particular for its neuroprotective effects, in several neurological disorders including ALS (for review see [27]) ***(*** ***Figure 5*** ***)***.

Glutamate excitotoxicity is considered to play an important role in pathological processes of motor neurons degeneration in amyotrophic lateral sclerosis [28]). In particular, a selective loss of excitatory amino-acid transporter 2 (EAAT2), a major glutamate transporter protein located on astrocytes and responsible of up to 94% of glutamate uptake at the synaptic cleft, has been described in ALS patients [29] and other neurodegenerative diseases [30], [31]. In mutant SOD1 mice, several studies have evidenced a decrease in EAAT2 protein associated with a down-regulation of glutamate-transport activity at late stage of the disease (for review [28]). Moreover, an over-expression of this transporter delays disease onset and prevents motoneurons death [32]. Interestingly, Da Cunha et al. have recently demonstrated in rats that moderate running-based exercise promotes glutamate uptake [33]. Motoneurons preservation and astrocytic modifications at P90 in the “sedentary treadmill”, 10 and 21 cm/s groups may result from a compensatory mechanism to overcome deficit in glutamate-transporter activity ***(*** ***Figure 5*** ***)***.

Besides increased astrogliosis, we also evidenced in the ventral horn of “sedentary treadmill” animals an increase in microglial reactivity. This finding is consistent with that of Williamson et al. that demonstrated Iba1 expression increase in the dentate gyrus of rats exposed to EE [34]. Ehninger et al. have reported a decrease of newborn microglia in the amygdala of healthy mice after EE exposure [35] but so far, the impact of EE on spinal cord microglia remains to be studied. Similarly, Vukovic et al. have shown that voluntary exercise leads to increased microglia number in the hippocampus of mice [36]. Microglia are recruited at neurodegeneration sites and are known to exert dual effects on neuronal survival notably by releasing pro- or anti- apoptotic factors [37], [38]. In particular, the CX_3_CL_1_-CX_3_CR_1_ pathway is known to provide a neuroprotective phenotype to microglia [37]. Moreover, soluble CX_3_CL_1_ is increased in the hippocampus after running exercise [36]. Thus, in our paradigm, enhanced glial reactivity may play a neuroprotective role that eventually contributes to better preservation and/or survival of motoneurons in “sedentary treadmill” and exercised mice ***(*** ***Figure 5*** ***)***.

To conclude, using a strictly controlled experimental protocols, we show that three intensities of running-based exercise failed to modify the lifespan of SOD1^G93A^ mice. However, exercise induces modifications at the cellular level in the spinal cord. We also show that conditions of housing/handling can significantly affect the life span of SOD1^G93A^ mice. This is of importance since it may not only explain previous outcome discrepancies reported in the literature on the effect of exercise in ALS but also, more generally, flawed results in the area of preclinical studies in CNS pathologies.

We demonstrate that a protracted survival induced by a slight daily modification of the environment is associated with motoneuron preservation and glial modifications in the lumbar spinal cord of SOD1^G93A^. Further studies are required to identify mechanisms involved in motoneuron preservation and survival and to decipher the contributions of glial cells in ALS pathogenesis.

## Materials and Methods

### Animals

Experimental procedures followed the European legislative, administrative and statutory measures for animal experimentation (86/609/EEC) and the Declaration of Helsinki. The study was approved by the “Direction des Services Vétérinaires de l'Hérault”, France (authorization number 34118) and ratified by the “Préfecture de l'Hérault”, France. Every effort was made to minimize the number of animals and their suffering. Transgenic mice carrying the G93A human SOD1 mutation B6SJL-Tg (SOD1-G93A) 1Gur/J (ALS mice) were purchased from The Jackson Laboratory (Bar Harbor, ME, USA) and bred on a B6SJL background. Transgenic mice were identified by PCR and housed in controlled conditions (hygrometry, temperature and 12 h light/dark cycle). Only males were used and litter-matching between groups and experiments were done as much as possible. A treadmill (Bioseb, LE8710, Chaville, France) was used to exercise the mice 15 min/day, 5 days a week. At day 60, males were isolated in individual cages (without any objects in their home cage) and randomly assigned to 5 different conditions: 2 sedentary groups [“sedentary” mice (n = 14) and “sedentary treadmill” mice placed on the inert treadmill (similar to trained mice but without any movement of the treadmill) (n = 13)], and 3 different training intensity groups 5 cm/s (n = 10), 10 cm/s (n = 10) and 21 cm/s (n = 10). Electrical shock system that encouraged the animals to run was disconnected to avoid bias due to stress. To accurately evaluate specific effects of the inert treadmill on the one hand and training intensities on the other hand, “sedentary” mice were handled similarly to other groups i.e. they were picked up by the tail and returned to their cage at the same time and frequency than other groups (5 days a week, see ***Figures S1 and S2***). All groups were tested between 9∶00 and 11∶00 am. To strictly follow the best practices and recommendations for preclinical studies using SOD1 mice, the age of death was recorded when mice were unable to right themselves 30s after having been pushed on their side [39]. To minimize as much as possible bias in the analysis of our data, we followed the recommendations of Benatar et al. [40], in particular regarding the number of animals. Moreover, all evaluations were done blind to the experimental conditions.

### Immunohistochemistry

Mice were anesthetized with pentobarbital (i.p), and perfused intracardially with cold PBS followed by cold 4% paraformaldehyde (Sigma Aldrich, Saint Louis, USA). Tissues were removed and post fixed for 24 h in 4% paraformaldehyde. Samples were cryoprotected in sucrose 30%, included in Tissue Teck (Sakura, Alphen aan den Rijn, Pays Bas), frozen and kept at −80° until processing.

Free floating spinal cord transverse sections (20 µm) were placed for 30 min in PBS containing lysine (20 mM, pH 7.4) and then for 15 min in 1% H_2_O_2._ Sections were then transferred for 1 hour in blocking solution (PBS, BSA (5%) and Triton X-100 (0.1%) (all from Sigma Aldrich, Saint Louis, USA)) and then incubated 48 hours at 4°C with either Iba1 (1/1000; Wako Pure Chemical Industries, Osaka, Japan) or GFAP (1/2000; Dako, Glostrup, Denmark) primary antibodies diluted in the same solution.

Secondary rabbit peroxydase-conjugated antibody was used (1∶500; Jackson Immunoresearch, Carlsbad, USA). Sections were then washed in TRIS buffer and enzymatic revelation was done with diaminobenzidine and H_2_0_2_ 0.1% as a substrate. Quantifications of the mean intensity labelling were done separately and blindly by two experimenters using the Adobe® Photoshop® software (Adobe, San Jose, USA).

Lumbar spinal cord sections were stained with Mayer hematoxylin solution for 15 minutes. Cells identified as motoneurons by their size, shape and location, were counted blindly for all groups. We quantified motoneurons soma areas using ImageJ v1.45 software (National Institutes of Health, Bethesda, MD, USA). Only motoneurons with an identifiable nucleus were included in the surface analysis. One section out of twenty (i.e. each 400 µm) was used all along the lumbar spinal cord segment.

### Neuromuscular Junctions Labelling

Neuromuscular junctions (NMJ) labelling was done following the protocol of Karnovsky and Roots [41]. We analyzed the entire *gastrocnemius-soleus-plantaris* muscular complex on transverse sections (16 µm). NMJ quantification was done blindly every 3^rd^ section.

### Statistical Analysis

Survival was analysed using Kaplan–Meier curves and log-rank test. For the quantification of glial cells and NMJ, comparisons between “sedentary” and “sedentary treadmill” animals were done using t-test (GraphPad Prism version 5.03, CA, USA). Experiments were designed to reach a 95% power to detect a 10% or greater difference between each exercise group and the sedentary treadmill group.

## Supporting Information

Figure S1 **“Sedentary treadmill” condition.** Mice are placed on an inert treadmill 15 minutes per day, 5 days a week.
(MP4)

Figure S2 **“Sedentary condition”.** Sedentary mice were handled similarly to other groups i.e. they were picked up by the tail and returned to their cage at the same time and frequency than other groups (5 days a week).
(MP4)

Figure S3 **Statistics. (Table A)** - Table of p-values for the survival analysis. **(Table B)** - Summary of p-values for motoneurons quantification. **(Table C)** - Table of p-values for Iba1 quantification. **(Table D)** - Table of p-values for GFAP quantification. * = significantly different as compared to the sedentary treadmill group (*, p<0.05; **, p<0.01; ***, p<0.001); # = significantly different as compared to the 5 cm/s group (#, p<0.05; ##, p<0.01); NS = non significant.
(DOC)

## References

[1] Ferraiuolo L, Kirby J, Grierson AJ, Sendtner M, Shaw PJ (2011) Molecular pathways of motor neuron injury in amyotrophic lateral sclerosis. Nat Rev Neurol 7: 616–630. 22051914 10.1038/nrneurol.2011.152

[2] Rothstein JD (2009) Current hypotheses for the underlying biology of amyotrophic lateral sclerosis. Ann Neurol 65 Suppl 1S3–9. 19191304 10.1002/ana.21543

[3] Boillee S, Yamanaka K, Lobsiger CS, Copeland NG, Jenkins NA, et al (2006) Onset and progression in inherited ALS determined by motor neurons and microglia. Science 312: 1389–1392. 16741123 10.1126/science.1123511

[4] Ferraiuolo L, Higginbottom A, Heath PR, Barber S, Greenald D, et al (2011) Dysregulation of astrocyte-motoneuron cross-talk in mutant superoxide dismutase 1-related amyotrophic lateral sclerosis. Brain 134: 2627–2641. 21908873 10.1093/brain/awr193 PMC3170534

[5] Philips T, Robberecht W (2011) Neuroinflammation in amyotrophic lateral sclerosis: role of glial activation in motor neuron disease. Lancet Neurol 10: 253–263. 21349440 10.1016/S1474-4422(11)70015-1

[6] Alexianu ME, Kozovska M, Appel SH (2001) Immune reactivity in a mouse model of familial ALS correlates with disease progression. Neurology 57: 1282–1289. 11591849 10.1212/wnl.57.7.1282

[7] Schiffer D, Cordera S, Cavalla P, Migheli A (1996) Reactive astrogliosis of the spinal cord in amyotrophic lateral sclerosis. J Neurol Sci 139 Suppl: 27–33 10.1016/0022-510x(96)00073-1 8899654

[8] Chio A, Benzi G, Dossena M, Mutani R, Mora G (2005) Severely increased risk of amyotrophic lateral sclerosis among Italian professional football players. Brain 128: 472–476. 15634730 10.1093/brain/awh373

[9] de Almeida JP, Silvestre R, Pinto AC, de Carvalho M (2012) Exercise and amyotrophic lateral sclerosis. Neurol Sci 33: 9–15. 22228269 10.1007/s10072-011-0921-9

[10] Patel BP, Hamadeh MJ (2009) Nutritional and exercise-based interventions in the treatment of amyotrophic lateral sclerosis. Clin Nutr 28: 604–617. 19782443 10.1016/j.clnu.2009.06.002

[11] Kaspar BK, Frost LM, Christian L, Umapathi P, Gage FH (2005) Synergy of insulin-like growth factor-1 and exercise in amyotrophic lateral sclerosis. Ann Neurol 57: 649–655. 15852403 10.1002/ana.20451

[12] Kirkinezos IG, Hernandez D, Bradley WG, Moraes CT (2003) Regular exercise is beneficial to a mouse model of amyotrophic lateral sclerosis. Ann Neurol 53: 804–807. 12783429 10.1002/ana.10597

[13] Grondard C, Biondi O, Armand AS, Lecolle S, Della Gaspera B, et al (2005) Regular exercise prolongs survival in a type 2 spinal muscular atrophy model mouse. J Neurosci 25: 7615–7622. 16107648 10.1523/JNEUROSCI.1245-05.2005 PMC6725405

[14] Ferrer-Alcon M, Winkler-Hirt C, Madani R, Perrin FE, Kato AC (2008) Low intensity exercise attenuates disease progression and stimulates cell proliferation in the spinal cord of a mouse model with progressive motor neuronopathy. Neuroscience 152: 291–295. 18295408 10.1016/j.neuroscience.2007.11.058

[15] Liebetanz D, Hagemann K, von Lewinski F, Kahler E, Paulus W (2004) Extensive exercise is not harmful in amyotrophic lateral sclerosis. Eur J Neurosci 20: 3115–3120. 15579165 10.1111/j.1460-9568.2004.03769.x

[16] Mahoney DJ, Rodriguez C, Devries M, Yasuda N, Tarnopolsky MA (2004) Effects of high-intensity endurance exercise training in the G93A mouse model of amyotrophic lateral sclerosis. Muscle Nerve 29: 656–662. 15116368 10.1002/mus.20004

[17] Fernando P, Bonen A, Hoffman-Goetz L (1993) Predicting submaximal oxygen consumption during treadmill running in mice. Can J Physiol Pharmacol 71: 854–857. 8143245 10.1139/y93-128

[18] Nithianantharajah J, Hannan AJ (2006) Enriched environments, experience-dependent plasticity and disorders of the nervous system. Nat Rev Neurosci 7: 697–709. 16924259 10.1038/nrn1970

[19] Stam NC, Nithianantharajah J, Howard ML, Atkin JD, Cheema SS, et al (2008) Sex-specific behavioural effects of environmental enrichment in a transgenic mouse model of amyotrophic lateral sclerosis. Eur J Neurosci 28: 717–723. 18702691 10.1111/j.1460-9568.2008.06374.x

[20] Deforges S, Branchu J, Biondi O, Grondard C, Pariset C, et al (2009) Motoneuron survival is promoted by specific exercise in a mouse model of amyotrophic lateral sclerosis. J Physiol 587: 3561–3572. 19491245 10.1113/jphysiol.2009.169748 PMC2742281

[21] Anastasia A, Torre L, de Erausquin GA, Masco DH (2009) Enriched environment protects the nigrostriatal dopaminergic system and induces astroglial reaction in the 6-OHDA rat model of Parkinson's disease. J Neurochem 109: 755–765. 19245661 10.1111/j.1471-4159.2009.06001.x PMC3575174

[22] Diniz DG, Foro CA, Rego CM, Gloria DA, de Oliveira FR, et al (2010) Environmental impoverishment and aging alter object recognition, spatial learning, and dentate gyrus astrocytes. Eur J Neurosci 32: 509–519. 20704596 10.1111/j.1460-9568.2010.07296.x

[23] Szeligo F, Leblond CP (1977) Response of the three main types of glial cells of cortex and corpus callosum in rats handled during suckling or exposed to enriched, control and impoverished environments following weaning. J Comp Neurol 172: 247–263. 838881 10.1002/cne.901720205

[24] Li J, Ding YH, Rafols JA, Lai Q, McAllister JP 2nd, et al (2005) Increased astrocyte proliferation in rats after running exercise. Neurosci Lett 386: 160–164. 16024173 10.1016/j.neulet.2005.06.009

[25] Gobbo OL, O'Mara SM (2004) Impact of enriched-environment housing on brain-derived neurotrophic factor and on cognitive performance after a transient global ischemia. Behav Brain Res 152: 231–241. 15196790 10.1016/j.bbr.2003.10.017

[26] Neeper SA, Gomez-Pinilla F, Choi J, Cotman CW (1996) Physical activity increases mRNA for brain-derived neurotrophic factor and nerve growth factor in rat brain. Brain Res 726: 49–56. 8836544

[27] Nagahara AH, Tuszynski MH (2011) Potential therapeutic uses of BDNF in neurological and psychiatric disorders. Nat Rev Drug Discov 10: 209–219. 21358740 10.1038/nrd3366

[28] Foran E, Trotti D (2009) Glutamate transporters and the excitotoxic path to motor neuron degeneration in amyotrophic lateral sclerosis. Antioxid Redox Signal 11: 1587–1602. 19413484 10.1089/ars.2009.2444 PMC2842587

[29] Rothstein JD, Van Kammen M, Levey AI, Martin LJ, Kuncl RW (1995) Selective loss of glial glutamate transporter GLT-1 in amyotrophic lateral sclerosis. Ann Neurol 38: 73–84. 7611729 10.1002/ana.410380114

[30] Behrens PF, Franz P, Woodman B, Lindenberg KS, Landwehrmeyer GB (2002) Impaired glutamate transport and glutamate-glutamine cycling: downstream effects of the Huntington mutation. Brain 125: 1908–1922. 12135980 10.1093/brain/awf180

[31] Li S, Mallory M, Alford M, Tanaka S, Masliah E (1997) Glutamate transporter alterations in Alzheimer disease are possibly associated with abnormal APP expression. J Neuropathol Exp Neurol 56: 901–911. 9258260 10.1097/00005072-199708000-00008

[32] Guo H, Lai L, Butchbach ME, Stockinger MP, Shan X, et al (2003) Increased expression of the glial glutamate transporter EAAT2 modulates excitotoxicity and delays the onset but not the outcome of ALS in mice. Hum Mol Genet 12: 2519–2532. 12915461 10.1093/hmg/ddg267

[33] da Cunha MJ, da Cunha AA, Ferreira AG, Machado FR, Schmitz F, et al (2012) Physical exercise reverses glutamate uptake and oxidative stress effects of chronic homocysteine administration in the rat. Int J Dev Neurosci 30: 69–74. 22244886 10.1016/j.ijdevneu.2012.01.001

[34] Williamson LL, Chao A, Bilbo SD (2012) Environmental enrichment alters glial antigen expression and neuroimmune function in the adult rat hippocampus. Brain Behav Immun. 10.1016/j.bbi.2012.01.003 PMC3294275 22281279

[35] Ehninger D, Wang LP, Klempin F, Romer B, Kettenmann H, et al (2011) Enriched environment and physical activity reduce microglia and influence the fate of NG2 cells in the amygdala of adult mice. Cell Tissue Res 345: 69–86. 21688212 10.1007/s00441-011-1200-z PMC3132349

[36] Vukovic J, Colditz MJ, Blackmore DG, Ruitenberg MJ, Bartlett PF (2012) Microglia modulate hippocampal neural precursor activity in response to exercise and aging. J Neurosci 32: 6435–6443. 22573666 10.1523/JNEUROSCI.5925-11.2012 PMC6621117

[37] Cardona AE, Pioro EP, Sasse ME, Kostenko V, Cardona SM, et al (2006) Control of microglial neurotoxicity by the fractalkine receptor. Nat Neurosci 9: 917–924. 16732273 10.1038/nn1715

[38] Sargsyan SA, Blackburn DJ, Barber SC, Grosskreutz J, De Vos KJ, et al (2011) A comparison of in vitro properties of resting SOD1 transgenic microglia reveals evidence of reduced neuroprotective function. BMC Neurosci 12: 91. 21943126 10.1186/1471-2202-12-91 PMC3191510

[39] Leitner M (2009) Working with ALS mice. Guidelines for preclinical testing and colony management.

[40] Benatar M (2007) Lost in translation: treatment trials in the SOD1 mouse and in human ALS. Neurobiol Dis 26: 1–13. 17300945 10.1016/j.nbd.2006.12.015

[41] Karnovsky MJ, Roots L (1964) A “Direct-Coloring” Thiocholine Method for Cholinesterases. J Histochem Cytochem 12: 219–221. 14187330 10.1177/12.3.219

