# Supplementary material for: Unlike Physical Exercise, Modified Environment Increases the Lifespan of SOD1G93A Mice However Both Conditions Induce Cellular Changes
Source: PLoS One. 2012 Sep 20;7(9):e45503. doi: 10.1371/journal.pone.0045503 (PMC3447796; doi:10.1371/journal.pone.0045503)
Supplement: Figure S3 — Statistics. (Table A) - Table of p-values for the survival analysis. (Table B) - Summary of p-values for motoneurons quantification. (Table C) - Table of p-values for Iba1 quantification. (Table D) - Table of p-values for GFAP quantification. * = significantly different as compared to the sedentary treadmill group (*, p<0.05; **, p<0.01; ***, p<0.001); # = significantly different as compared to the 5 cm/s group (#, p<0.05; ##, p<0.01); NS = non significant. (DOC) [file pone.0045503.s003.doc]

***Supplemental Table A: Summary of p-values for the survival curves***

| **SedT vs Sed** | **SedT vs 5cm/s** | **SedT vs 10cm/s** | **SedT vs 21cm/s** | **5cm/s vs 10cm/s** | **5cm/s vs 21cm/s** | **10cm/s vs 21cm/s** |
| --- | --- | --- | --- | --- | --- | --- |
| **0.0015 **** | 0.0745 | 0.3209 | 0.8228 | 0.5659 | **0.0439 *** | 0.2627 |

*= significantly different as compared to the sedentary treadmill group (*, p<0.05; **, p<0.01)

***Supplemental Table B: Summary of p-values for motoneurons quantifications***

|  |  | **SedT vs Sed** | **SedT vs 5cm/s** | **SedT vs 10cm/s** | **SedT vs 21cm/s** | **5cm/s vs 10cm/s** | **5cm/s vs 21cm/s** | **10cm/s vs 21cm/s** |
| --- | --- | --- | --- | --- | --- | --- | --- | --- |
|  | **Number** | **0.0053 **** | 0.5092 | **0.0132 *** | **0.0486 *** | **0.0429 *** | 0.1289 | 0.5370 |
| **Mean Area** | **<300µm** | 0.4668 | **0.0263 *** | 0.3387 | **0.0181 *** | 0.7457 | 0.0581 | 0.3216 |
|  | **300-500µm** | 0.3301 | **0.0206 *** | 0.3462 | 0.1401 | 0.7459 | 0.7287 | 0.6382 |
|  | **500-700µm** | **0.0006 ***** | 0.0764 | 0.2104 | 0.1063 | 0.9071 | **0.0281 *** | 0.0666 |
|  | **700-900µm** | 0.3163 | **0.0129 *** | 0.0576 | **0.0326 *** | 0.5894 | 0.4077 | 0.8535 |
|  | **>900µm** | **0.0016 **** | **0.0055 **** | 0.1707 | **0.0351 *** | 0.7870 | 0.8859 | 0.7491 |

*= significantly different as compared to the sedentary treadmill group (*, p<0.05; **, p<0.01; ***, p<0.001)

***Supplemental Table C: Summary of p-values*** for Iba1 quantification

| **Comparison** | **Total section** | **Dorsal spinal cord** | **Ventral spinal cord** | **White matter** | **Grey matter** |
| --- | --- | --- | --- | --- | --- |
| **SedT vs Sed** | **0.0140 *** | 0.0631 NS | **0.0058 **** | **0.0482 *** | **0.0087 **** |
| **SedT vs 5cm/s** | 0.0524 NS | 0.1440 NS | **0.0148 *** | 0.1265 NS | **0.0349 *** |
| **SedT vs 10cm/s** | 0.1606 NS | 0.0680 NS | 0.5610 NS | 0.1888 NS | 0.1862 NS |
| **SedT vs 21cm/s** | 0.2550 NS | 0.1034 NS | 0.8589 NS | 0.3019 NS | 0.2697 NS |
| **5cm/s vs 10cm/s** | 0.2083 NS | 0.7237 NS | 0.0640 NS | 0.6955 NS | 0.0535 NS |
| **5cm/s vs 21cm/s** | 0.1504 NS | 0.8785 NS | **0.0386 #** | 0.4298 NS | 0.0647 NS |
| **10cm/s vs 21cm/s** | 0.6276 NS | 0.8256 NS | 0.7235 NS | 0,6568 NS | 0.6658 NS |

*= significantly different as compared to the sedentary treadmill group (*, p<0.05; **, p<0.01); # = significantly different as compared to the 5cm/s group (#, p<0.05; ##, p<0.01); NS= non significant

***Supplemental Table D***: Summary of p-values for GFAP quantification

| **Comparison** | **Total section** | **Dorsal spinal cord** | **Ventral spinal cord** | **White matter** | **Grey matter** |
| --- | --- | --- | --- | --- | --- |
| **SedT vs Sed** | **0.0197 *** | 0.2501 NS | **0.0021 **** | 0.5303 NS | **0.0126 *** |
| **SedT vs 5cm/s** | **0.0044 **** | **0.0232 *** | **0.0018 **** | **0.0178 *** | **0.0049 **** |
| **SedT vs 10cm/s** | 0.0690 NS | **0.0389 *** | 0.1989 NS | 0.0581 NS | 0.1744 NS |
| **SedT vs 21cm/s** | 0.3549 NS | 0.1344 NS | 0.8913 NS | 0.6788 NS | 0.3092 NS |
| **5cm/s vs 10cm/s** | **0.0260 #** | 0.4515 NS | **0.0098 ##** | 0.1431 NS | **0.0117 #** |
| **5cm/s vs 21cm/s** | **0.0111  #** | 0.1059 NS | **0.0079 ##** | **0.0211 #** | **0.0081 ##** |
| **10cm/s vs 21cm/s** | 0.1710  NS | 0.2033 NS | 0.3390 NS | **0,0454 *** | 0.5043 NS |

*= significantly different as compared to the sedentary treadmill group (*, p<0.05; **, p<0.01); # = significantly different as compared to the 5cm/s group (#, p<0.05; ##, p<0.01); NS= non significant
